# Solving the Inverse Problem of Electrocardiography on the Endocardium Using a Single Layer Source

**DOI:** 10.3389/fphys.2019.00058

**Published:** 2019-02-05

**Authors:** Alexander Kalinin, Danila Potyagaylo, Vitaly Kalinin

**Affiliations:** EP Solutions SA, Yverdon-les-Bains, Switzerland

**Keywords:** inverse ECG problem, transfer matrix, Tikhonov regularization, single layer potential, endocardial surface

## Abstract

The inverse problem of electrocardiography consists in reconstructing cardiac electrical activity from given body surface electrocardiographic measurements. Despite tremendous progress in the field over the last decades, the solution of this problem in terms of electrical potentials on both epi- and the endocardial heart surfaces with acceptable accuracy remains challenging. This paper presents a novel numerical approach aimed at improving the solution quality on the endocardium. Our method exploits the solution representation in the form of electrical single layer densities on the myocardial surface. We demonstrate that this representation brings twofold benefits: first, the inverse problem can be solved for the physiologically meaningful single layer densities. Secondly, a conventional transfer matrix for electrical potentials can be split into two parts, one of which turned out to posess regularizing properties leading to improved endocardial reconstructions. The method was tested *in-silico* for ventricular pacings utilizing realistic CT-based heart and torso geometries. The proposed approach provided more accurate solution on the ventricular endocardium compared to the conventional potential-based solutions with Tikhonov regularization of the 0th, 1st, and 2nd orders. Furthermore, we show a uniform spatio-temporal behavior of the single layer densities over the heart surface, which could be conveniently employed in the regularization procedure.

## 1. Introduction

Non-invasive electrocardiographic imaging (ECGI) is a novel imaging modality which is based on numerical reconstruction of cardiac electrical activity using the so-called body surface potential maps (BSPM) and patient-specific heart and torso geometries (Ramanathan et al., 2004). The ultimate goal of the ECGI is to allow non-invasive panoramic cardiac mapping in a beat-to-beat mode, thus facilitating diagnostics and treatment planning for non-sustained, aperiodic or non-tolerable cardiac arrhythmia.

A mathematical problem underlaying ECGI is known as the inverse problem of ECG. This problem can be formulated in several ways with respect to the unknown physical values that are sought after. Its early formulation concerned pericardial (also called epicardial) potentials, which by definition neglect electrophysiological processes ongoing on the inner heart walls (Franzone et al., 1978; Rudy and Messinger-Rapport, 1988). Mathematically, this is a Cauchy problem for the Laplace equation, a classical example of ill-posed problem: even small amount of noise in the Cauchy boundary data can lead to arbitrary high errors in the solution (Kubo, 1994; Takeuchi and Yamamoto, 2008). Therefore, special regularizing algorithms must be used in order to obtain a stable solution approximation.

With this respect, significant progress has been recently made in the development of numerical algorithms for solving the inverse problem of ECG in terms of epicardial potentials, which is reflected in a constantly increasing number of clinical applications of the ECGI. This methodology was successfully used for optimizing cardiac resynchronization therapy, guiding catheter ablation of origins of focal atrial and ventricular tachycardia, detecting macro-reentrant circuits and electrical rotors in patients with reentrant ventricular tachycardia, atrial flutter and atrial fibrillation (Guillem et al., 2013; Erkapic et al., 2014; Shah et al., 2014; Dubois et al., 2015; Varma, 2015; Rodrigo et al., 2017). Moreover, Cuculich et al. demonstrated the way ECGI can provide a support for guiding non-invasive ablation of cardiac arrhythmia (Cuculich et al., 2017).

Unfortunately, epicardial imaging bears one significant limitation of potentially losing electrophysiologically relevant information about cardiac electrical activity on the cardiac endocardium and, especially, on the interventricular and interatrial septum. To overcome this drawback, one can consider reconstructing electrical potentials on both epicardial and endocardial heart surfaces. Formally, this statement results in the same Cauchy problem for the Laplace equation. Due to a more complex non-convex geometry of the epi-endocardial heart surface compared to its relatively simple “convex hull” (epicardium/pericardium), the inverse problem for endo-epicardial reconstruction becomes even worse conditioned.

Nevertheless, solution of the endo-epicadial inverse problem was employed for detection of origins of focal ventricular tachycardia (Revishvili et al., 2015; Wissner et al., 2016), determination of electrical rotors in atrial fibrillation (Metzner et al., 2017), exploring morphology of unipolar epicardial and endocardial electrograms in the right ventricular outflow tract in patients with Brugada syndrome (Rudic et al., 2016), analysis of excitation patterns in reentrant ventricular tachycardia (Tsyganov et al., 2017) and atrial flutter (Wissner et al., 2018). In these studies, a numerical algorithm based on a combination of Tikhonov and iterative regularization was used (Bokeriya et al., 2008; Kalinin, 2011).

Alternative to the potential-based statement, the problem can also be formulated in terms of surface electrical layer source models. The most prominent example of such statement is the equivalent double layer (EDL) defined on both epi- and endocardial surfaces of the heart (van Oosterom, 2014). According to the bidomain model (Tung, 1978), the EDL is proportional to the transmembrane potential when the body electrical conductivity as well as the extracellular and intracellular myocardial conductivities are considered to be isotropic and the sum of the extracellular and intracellular conductivities is equal to those of the body (Geselowitz, 1989; Kalinin et al., 2017). This electrophysiological meaning was shown to be highly beneficial for construction of ECGI-specific regularization techniques (Berger et al., 2006, 2011; van Dam et al., 2009).

In contrast to the surface EDL source model, the electrical sources inside the myocardium cannot be reconstructed unambiguously (Geselowitz, 1989; Kalinin et al., 2017). However, employement of proper regularization schemes targeting intramural transmembrane potentials or current densities was reported to overcome this rather theoretical limitation delivering promising results (see for example He et al., 2003; Skipa, 2004; Schulze et al., 2013; Wang et al., 2013; Xu et al., 2014; Zhou et al., 2016).

Overall, despite the efforts and progress made in the ECGI field, non-invasive reconstruction of the local cardiac activity on both epi- and endocardium of the heart remains a challenging task for clinical, mathematical and engineering research. Furthermore, it is evident that, irrespectively of the source model under consideration, effectiveness of Tikhonov regularization method strongly depends on the choice of a regularization operator *R*. In addition to the simplest option, i.e., using an identity matrix, the surface Laplacian *L* as well as an operator *D* mapping the electrical potential on the cardiac surface to its normal derivative or the transmural gradient were used for the ECGI applications (Horácek and Clements, 1997; Erem et al., 2014; Wang et al., 2016). However, the problem of an optimal choice of the regularization operator in Tikhonov regularization is still open.

In this article, we describe a novel numerical approach for treating the epi-endocardial reconstruction problem by introducing an alternative source model formulation, the single layer density. We consider this problem from three interrelated perspectives. From the numerical algebraic point of view, we introduce the involved transfer matrices associated to the boundary elements method. Furthermore, we investigate regularizing properties of the inverse single layer operator for Tikhonov regularization. Finally, we investigate spatio-temporal behavior of the single layer density source model, which can be employed in the regularization procedures.

## 2. Methods

**Figure 1:**
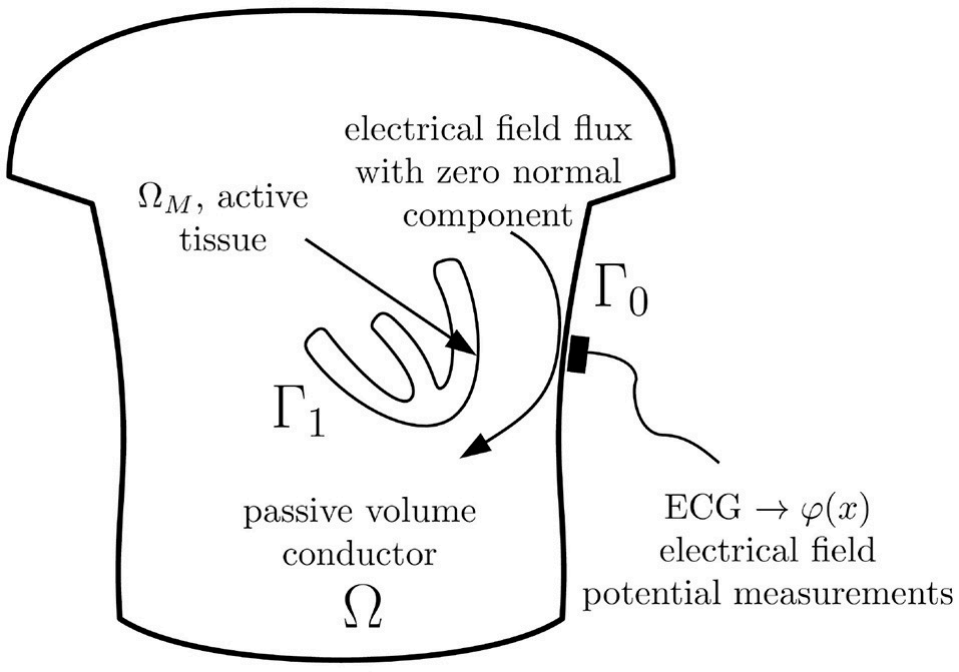
Physical model underlying the inverse ECG problem.

In this paper we use the geometry notation reported in Figure 1. Let Γ_0_ be a body surface and Γ_1_ be a surface of ventricles (or atria) circumpassing both epi- and endocardial parts. Let Ω ⊂ ℝ^3^ be a body domain bounded from the outside by the surface Γ_0_ and from the inside by surface Γ_1_, with outward unit normal vectors. Let Ω_*M*_ ⊂ Ω represent the myocardial domain bounded by Γ_1_ with inward unit normal vectors. Surfaces Γ_0_ and Γ_1_ are supposed to be sufficiently smooth.

In the physical model considered here, the electrical field is originated by the electrical sources situated in the myocardium domain Ω_*M*_ only. We neglect electrical sources in the human body domain Ω and consider the body domain as a passive volume conductor. This body domain Ω includes extracardiac organs as well as the cardiac chambers filled with the blood. Moreover, for those time moments when the ventricles/atria are in the resting state the atrial/ventricular myocardium can be also considered as a passive volume conductor.

The commonly used approach for the mathematical description of myocardial electrical activity is based on so the called bidomain model. Within this model, myocardial tissue is considered to consist of homogenized intracellular and extracellular spaces. The set of accompanying differential equations establishes the relationship between the intra- and extracellular potentials taking into account cell membrane properties and ionic concentrations (see e.g., Tung, 1978; Bourgault et al., 2009). The electrical conductivity in the intra- and extracellular media are provided in tensor form reflecting faster excitation propagation along the myocardial fibers than across them. In this work, we employed the bidomain model with isotropic cardiac electrical conductivity values and a homogeneous torso model to simulate the electrical potentials throughout the geometry volume for ectopic ventricular stimuli. The potentials obtained on the body surface, the body surface potential maps (BSPM), were then used as the input for validating the proposed approach of non-invasive reconstruction of cardiac electrical activity. This, so-called forward problem of ECG, was solved with the CHASTE software (Mirams et al., 2013), which provided the reference transmembrane potentials in the heart and electrical potentials in the whole geometry volume.

For biological tissues frequencies under the conventional assumptions the Maxwell equations, describing propagation of electromagnetic fields in the body volume conductor, can be simplified to the quasi-static form (Gulrajani, 1998). It allows temporal separation of the cardiac sources, meaning that one can solve the inverse problem of ECG for distinct time instants *t*_0_, *t*_1_, …, *t*_*M*_ independently from each other.

Furthermore, the inverse problem of ECG can be treated using direct and indirect regularization approaches. The direct way is the computation of the harmonic function value on Γ_1_ without considering myocardial electrical sources. The indirect way consists of presenting the electrical potential on Γ_1_ as a potential of the myocardial sources. It is well known that an endless number of the sources distributions in the myocardium domain can generate the same potential in the passive volume conductor domain. Therefore, they use an “effective” unique representation of the electrical sources in form of sources on the myocardial surface. In this article we consider the direct and the indirect ways for numerical solving the inverse electrocardiography problem.

### 2.1. Computational Method for the Inverse Potential Problem: A Conventional Approach

**Figure 2:**
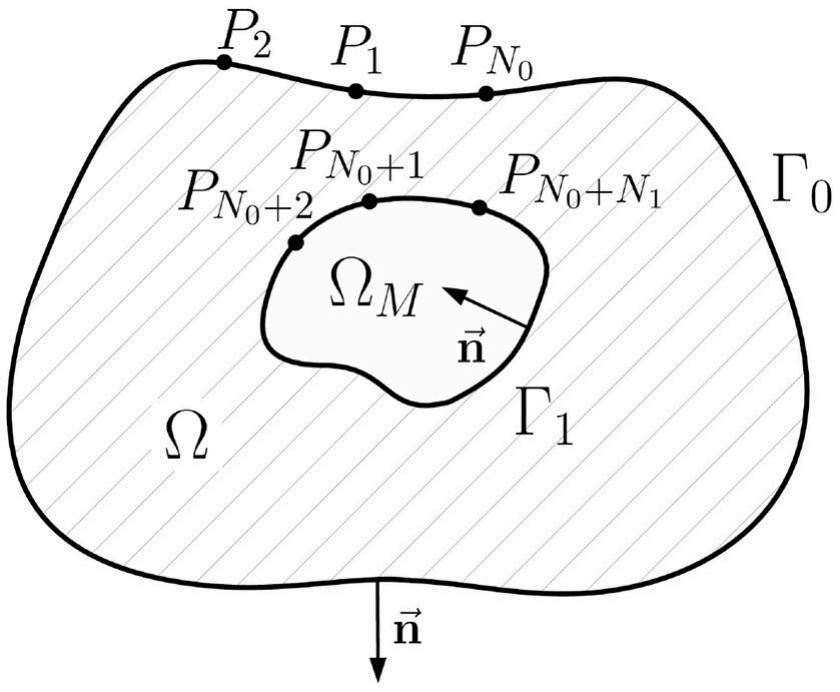
Schematic geometric relationships of the inverse potential problem in the internal statement. Ω is the passive volume conductor domain, Ω_*M*_ is the myocardial domain, Γ_0_ is the body surface, Γ_1_ is the myocardial surface (endo- and epicardial surface), $\vec{\text{n}}$ its unit normal vector directed inward, *P*_*i*_, *i* = 1..*N*_0_+*N*_1_ are collocation points used in direct boundary element method, *N*_0_ is the number of collocation points on the Γ_0_, *N*_1_ is the number of collocation points on Γ_1_.

The inverse problem of ECG in terms of electrical potentials for the geometry depicted in Figure 2 reads to find a function *u*(*x*) in $\bar{\Omega }$ such that

(1) $$\begin{array}{r} \Delta u\left(x\right)=0,\;x\in \Omega , \end{array}$$

(2) $$\begin{array}{r} u\left(x\right)=\varphi \left(x\right),\;x\in {\text{Γ}}_{0}, \end{array}$$

(3) $$\begin{array}{r} \frac{\partial u\left(x\right)}{\partial n}=0,\;x\in {\text{Γ}}_{0}, \end{array}$$

where φ(*x*) is the measured BSPM. Problem (1)–(3) is known as the Cauchy problem for the Laplace equation. Its solution is unique, however, the problem is ill-posed: even a small amount of noise in the boundary conditions can lead to an arbitrary high error in the solution.

To solve the problem a direct boundary element method (BEM) can be used. Accordingly, the problem (1)–(3) is reformulated as a boundary integral equation based on the third Green's identity: for any given point *P*∈Γ_0_∪Γ_1_ and harmonic function *u* in domain Ω

(4) $$\begin{array}{r} c\left(P\right)u\left(P\right)+\underset{{\text{Γ}}_{0}\cup {\text{Γ}}_{1}}{\int }u\left(Q\right)\frac{\partial G\left(P,Q\right)}{\partial n}d\text{Γ}=\underset{{\text{Γ}}_{0}\cup {\text{Γ}}_{1}}{\int }\frac{\partial u\left(Q\right)}{\partial n}G\left(P,Q\right)d\text{Γ}, \end{array}$$

where *Q* ∈ *dΓ* is the integration variable and

(5) $$\begin{array}{r} G\left(P,Q\right)=\frac{1}{\left|P-Q\right|} \end{array}$$

is the inverse Euclidean distance between *P* and *Q*, *c*(*P*) is the solid angle at the point *P*.

Next step is to establish a system of linear algebra equations suitable for numerical calculations from the continues statement (4). This step is called discretization. We use the following discretization scheme: (a) approximation of the surfaces Γ_0_ and Γ_1_ by the triangular meshes, (b) approximation of the functions *u*(*x*) and $\frac{\partial u\left(x\right)}{\partial n}$ by series of linear basis functions, and (c) computation of the single and double layer type integrals over basis functions. Computation of such integrals over basis function is most important step. Fortunately, it is well studied, see e.g., Dunavant (1985), Davey and Hinduja (1989), and van Oosterom (2012). Full details of the discretization process are described in the Appendix A.

After the discretization we get the following system of linear equations:

(6) $$\begin{array}{r} \begin{array}{c} H_{00}u_{0}+H_{01}u_{1}=G_{01}q_{1}\\ H_{10}u_{0}+H_{11}u_{1}=G_{11}q_{1} \end{array} \end{array}$$

where *u*_0_, *u*_1_ are electrical potentials on the surfaces Γ_0_ and Γ_1_ respectively, *q*_1_ is the normal derivative of the electrical potential on the surface Γ_1_, matrices *G*_*ij*_ arise from the discretization of the surface integrals corresponding to the single layer

(7) $$\begin{array}{r} \underset{{\text{Γ}}_{j}}{\int }\frac{\partial u\left(Q\right)}{\partial n}G\left(P_{{\text{Γ}}_{i}},Q\right)d{\text{Γ}}_{Q}, \end{array}$$

while matrices *H*_*ij*_ arise from the discretization of the surface integrals corresponding to the double layer

(8) $$\begin{array}{r} \underset{{\text{Γ}}_{j}}{\int }u\left(Q\right)\frac{\partial G\left(P_{{\text{Γ}}_{i}},Q\right)}{\partial n}d{\text{Γ}}_{Q}, \end{array}$$

Finally, *i* is the index of the surface containing the point *P*, *j* is that of the surface containing *Q*.

**Figure 3:**
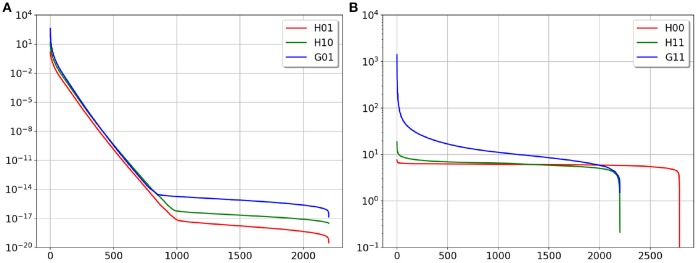
SVD plot of the considered matrices, **(A)** is ill-conditioned matrices, **(B)** is well-conditioned matrices.

In Figure 3 we provide the plots of the singular values' decay of the matrices above. In agreement with the boundary element theory, matrices *G*_11_, *H*_00_ and *H*_11_ are well-conditioned and can be inverted without regularization.

From the system (6) we can get the transfer matrix *u*_1_→*u*_0_ (relating EP to BSPM, EP stands for endo- and epicardial potentials):

(9) $$\begin{array}{r} \left(-H_{01}+G_{01}G_{11}^{-1}H_{11}\right)u_{1}=\left(H_{00}-G_{01}G_{11}^{-1}H_{10}\right)u_{0}, \end{array}$$

in short form

(10) $$\begin{array}{r} Au_{1}=f_{0}, \end{array}$$

where *f*_0_ is known right-hand side of Equation (9). In the following, we call the inverse ECG problem statement (1)–(3) the *internal* statement and Equation (9) the *internal* equation.

In order to find the normal derivative of the potential on Γ_1_ let us introduce the Dirichlet-Neumann mapping matrix *u*_1_→*q*_1_ relating EP to its normal derivative on the heart. This matrix can be derived from the system (6) in the form

(11) $$\begin{array}{r} {\left(G_{11}-H_{10}H_{00}^{-1}G_{01}\right)}^{-1}\left(H_{11}-H_{10}H_{00}^{-1}H_{01}\right)u_{1}=q_{1}, \end{array}$$

or

(12) $$\begin{array}{r} Du_{1}=q_{1} \end{array}$$

Note that representations (9) and (11) require inversion of well-conditioned matrices only. Matrices *A* and *D* are well known in the literature (e.g., see Yun et al., 1997; Gulrajani, 1998).

Matrix *A* is ill-conditioned, therefore the numerical solution of Equation (10) requires suitable regularization techniques. The commonly used approach is the Tikhonov regularization method:

(13) $$\begin{array}{r} u_{1}^{\lambda }=\text{arg min}\left(∥Au_{1}-f_{0}∥{}_{2}^{2}+\lambda^{2}∥Ru_{1}∥{}_{2}^{2}\right), \end{array}$$

where $u_{1}^{\lambda }$ is the regularized solution, λ^2^ is the regularization parameter and *R* is the regularization operator. Minimization problem (13) has the closed-form solution:

(14) $$\begin{array}{r} u_{1}^{\lambda }={\left(A^{T}A+\lambda^{2}R^{T}R\right)}^{-1}A^{T}f_{0}. \end{array}$$

Regularization operator *R* can be taken, for example, as *R* = *I*_11_ (identity matrix) for 0 order, *R* = *D* for 1th order and as a Laplace-Beltrami (“surface Laplacian”) operator *Lu*_1_ = Δ_Γ_1__*u*_1_ (see for example Huiskamp, 1991) for the 2nd order Tikhonov regularization.

### 2.2. Computational Method for the Inverse Potential Problem: A Single Layer Approach

**Figure 4:**
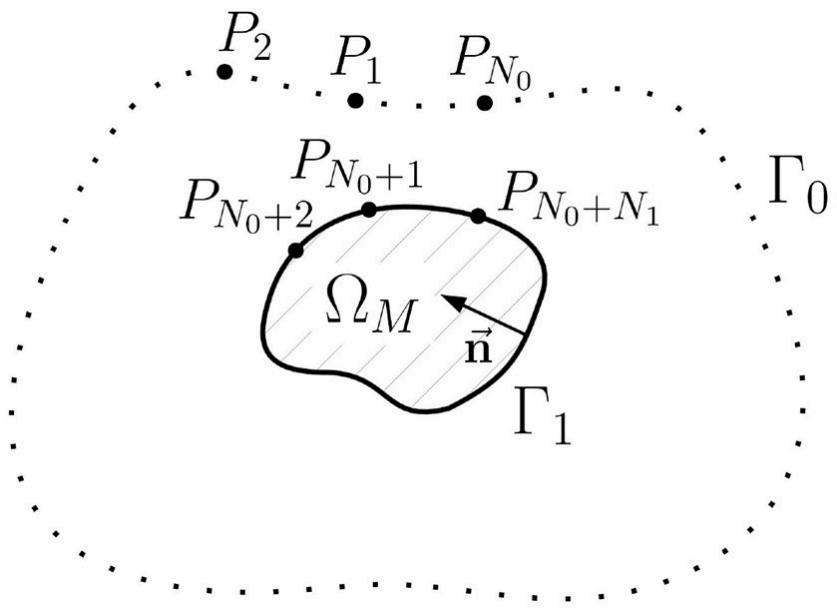
Schematic geometric relationships of the inverse potential problem in the external statement. Ω_*M*_ is the myocardial domain, Γ_0_ is the body surface, Γ_1_ is the myocardial surface (endo- and epicardial surface), $\vec{\text{n}}$ is unit normal vector, *P*_*i*_, *i* = 1..*N*_0_+*N*_1_ are collocation points used in direct boundary element method, *N*_0_ is the number of collocation points on the Γ_0_, *N*_1_ is the number of collocation points on Γ_1_.

In this section we will formulate an alternative representation of the *u*_1_→*u*_0_ transfer matrix and propose a new statement of the inverse problem in terms of the equivalent single layer (ESL). Geometry notations for this statement are depicted on Figure 4.

Although the cardiac electrical potential *u*(*x*) is not a harmonic function in the domain Ω_*M*_, we can define another function û harmonic in Ω_*M*_ whose boundary values on Γ_1_ are equal to those of *u*(*x*), the solution of problem (1)–(3). The function û can be defined as the unique solution of the following Dirichlet problem for the Laplace equation:

(15) $$\begin{array}{r} \Delta û\left(x\right)=0,\;x\in \Omega_{M}, \end{array}$$

(16) $$\begin{array}{r} û\left(x\right)=u\left(x\right),\;x\in {\text{Γ}}_{1}. \end{array}$$

The idea under this definition is following. With the function û(*x*) harmonic in the domain Ω_*M*_ we force the electric sources to be only on the endo- and epicardial surface. And with the condition û(*x*) = *u*(*x*) on Γ_1_ we can link such sources densities with the actual electrical potential *u*(*x*). To derive such relationships we use boundary element method.

For any given point *P* ∈ Γ_1_ and harmonic function û in domain Ω_*M*_ the third Green's identity gives us the following boundary integral equation

(17) $$\begin{array}{l} ĉ\left(P\right)û\left(P\right)+\underset{{\text{Γ}}_{1}}{\int }û\left(Q\right)\frac{\partial G\left(P,Q\right)}{\partial n}d{\text{Γ}}_{Q}\\ \;=\underset{{\text{Γ}}_{1}}{\int }\frac{\partial û\left(Q\right)}{\partial n}G\left(P,Q\right)d{\text{Γ}}_{Q},\;P\in {\text{Γ}}_{1}. \end{array}$$

Moreover, for any point *P* ∈ Γ_0_ and harmonic function û in domain Ω_*M*_ the third Green's identity give us the following boundary integral equation

(18) $$\begin{array}{r} \underset{{\text{Γ}}_{1}}{\int }û\left(Q\right)\frac{\partial G\left(P,Q\right)}{\partial n}d{\text{Γ}}_{Q}=\underset{{\text{Γ}}_{1}}{\int }\frac{\partial û\left(Q\right)}{\partial n}G\left(P,Q\right)d{\text{Γ}}_{Q},\;P\in {\text{Γ}}_{0}, \end{array}$$

Applying the same discretization as in section 2.1 we get the following algebraic system:

(19) $$\begin{array}{r} \begin{array}{c} {Ĥ}_{11}{û}_{1}={Ĝ}_{11}{\hat{q}}_{1}\\ {Ĥ}_{01}{û}_{1}={Ĝ}_{01}{\hat{q}}_{1}. \end{array} \end{array}$$

where û_1_ is a vector containing values of function û(*x*) at the points on the surface Γ_1_, ${\hat{q}}_{1}$ is a vector containing values of function $\frac{\partial û\left(x\right)}{\partial n}$ on Γ_1_, matrices Ĝ_*ij*_ arise from the discretization of the surface integrals corresponding to the single layer

(20) $$\begin{array}{r} \underset{{\text{Γ}}_{j}}{\int }\frac{\partial û\left(Q\right)}{\partial n}G\left(P_{{\text{Γ}}_{i}},Q\right)d{\text{Γ}}_{Q}, \end{array}$$

matrices Ĥ_*ij*_ arise from the discretization of the surface integrals corresponding to the double layer

(21) $$\begin{array}{r} \underset{{\text{Γ}}_{j}}{\int }û\left(Q\right)\frac{\partial G\left(P_{{\text{Γ}}_{i}},Q\right)}{\partial n}d{\text{Γ}}_{Q}, \end{array}$$

*i* is the index of the surface with the fixed point *P*, *j* is the index of the surface with points of integration *Q*.

We can express the unknown variable ${\hat{q}}_{1}$ from the first equation and obtain a new matrix-vector identity for the variable û_1_

(22) $$\begin{array}{r} {Ĥ}_{01}{û}_{1}={Ĝ}_{01}{Ĝ}_{11}^{-1}{Ĥ}_{11}{û}_{1} \end{array}$$

Using (16) we can write (22) as

(23) $$\begin{array}{r} {Ĥ}_{01}u_{1}={Ĝ}_{01}{Ĝ}_{11}^{-1}{Ĥ}_{11}u_{1}. \end{array}$$

Let us compare matrices Ĝ_01_, Ĝ_11_, Ĥ_01_, and Ĥ_11_ with matrices *G*_01_, *G*_11_, *H*_01_, and *H*_11_ defined in section 2.1. All matrices are determined only by the same surfaces Γ_0_ and Γ_1_. However, normal vectors to the surface Γ_1_ were directed outwards related to the domain Ω and inwards related to the domain Ω_*M*_. Taking into account these facts it is easy to demonstrate that

(24) $$\begin{array}{r} {Ĝ}_{01}=G_{01}, \end{array}$$

(25) $$\begin{array}{r} {Ĝ}_{11}=G_{11}, \end{array}$$

(26) $$\begin{array}{r} {Ĥ}_{01}=H_{01}, \end{array}$$

(27) $$\begin{array}{r} {Ĥ}_{11}=H_{11}-4\pi I_{11}, \end{array}$$

where *I*_11_ is the identity matrix.

Now we can rewrite (23) using the matrix defined in section 2.1:

(28) $$\begin{array}{r} H_{01}u_{1}=G_{01}G_{11}^{-1}\left(H_{11}-4\pi I_{11}\right)u_{1} \end{array}$$

or

(29) $$\begin{array}{r} 4\pi G_{01}G_{11}^{-1}u_{1}=\left(-H_{01}+G_{01}G_{11}^{-1}H_{11}\right)u_{1} \end{array}$$

Comparing (29) with (9) we can see that the right hand side of the Equation (29) is the same as the left hand side of the Equation (9). Therefore

(30) $$\begin{array}{r} 4\pi G_{01}G_{11}^{-1}u_{1}=\left(H_{00}-G_{01}G_{11}^{-1}H_{10}\right)u_{0} \end{array}$$

or in short form

(31) $$\begin{array}{r} 4\pi G_{01}G_{11}^{-1}u_{1}=f_{0}. \end{array}$$

Equation (31) is a new representation of the *u*_1_→*u*_0_ transfer matrix. In this article we call this approach the *external* statement of the inverse problem and transfer matrix (30) the *external* form of the transfer matrix.

Matrix *G*_11_ is well-conditioned (see Figure 3), so we can define a new function

(32) $$\begin{array}{r} w_{1}\equiv G_{11}^{-1}u_{1} \end{array}$$

and new equation for the function *w*_1_

(33) $$\begin{array}{r} 4\pi G_{01}w_{1}=f_{0}. \end{array}$$

Matrices *G*_11_ and *G*_01_ are discretizations of the single layer integral operators. Therefore, the function *w*_1_ has a physical meaning of electrical sources in form of single layer on the myocardial surface. In this paper we call function *w*_1_ the equivalent single layer (ESL) density. Equation (33) allows us to solve the inverse ECG problem in terms of the ESL.

Furthermore, we propose two methods for regularizing the inverse potential problem. The first method consists of the ESL computation by solving the Equation (33) and reconstruction of the potential *u*_1_ from the obtained ESL by formula *u*_1_ = *G*_11_*w*_1_.

Matrix *G*_01_ is ill-conditioned, therefore the numerical solution of Equation (33) requires suitable regularization algorithms. The Tikhonov regularization method of 0th order consists in solving

(34) $$\begin{array}{r} w_{1}^{\lambda }=\text{arg min}\left(4\pi ∥G_{01}w_{1}-f_{0}∥{}_{2}^{2}+\lambda^{2}∥w_{1}∥{}_{2}^{2}\right), \end{array}$$

whose solution reads

(35) $$\begin{array}{r} w_{1}^{\lambda }={\left(G_{01}^{T}G_{01}+\lambda^{2}I_{11}\right)}^{-1}G_{01}^{T}f_{0}, \end{array}$$

and next we compute

(36) $$\begin{array}{r} u_{1}^{\lambda }=G_{11}w_{1}^{\lambda }, \end{array}$$

where $w_{1}^{\lambda }$, $u_{1}^{\lambda }$ are the regularized solutions in terms of the ESL and potentials respectively, λ^2^ is the regularization parameter.

The second method relies on solving the transfer Equation (10) using Tikhonov regularization method with constraints on the ESL, i.e., using inverse ESL matrix $G_{11}^{-1}$ as regularization operator:

(37) $$\begin{array}{r} u_{1}^{\lambda }=\text{arg min}\left(∥Au_{1}-f_{0}∥{}_{2}^{2}+\lambda^{2}∥w_{1}∥{}_{2}^{2}\right), \end{array}$$

or

(38) $$\begin{array}{r} u_{1}^{\lambda }=\text{arg min}\left(∥Au_{1}-f_{0}∥{}_{2}^{2}+\lambda^{2}∥G_{11}^{-1}u_{1}∥{}_{2}^{2}\right), \end{array}$$

with solution:

(39) $$\begin{array}{r} u_{1}^{\lambda }={\left(A^{T}A+\lambda^{2}{G_{11}^{-1}}^{T}G_{11}^{-1}\right)}^{-1}A^{T}f_{0}. \end{array}$$

### 2.3. Experimental Methods and Evaluation Protocols

Accuracy of the numerical algorithms for solving the inverse problem of ECG was tested on realistic *in-silico* data of cardiac electrical activity. Computer tomography (CT) scans of three patients were used for obtaining the personalized anatomy of the torso and heart surfaces.

These patients were examined in Bakulev National Medical Research Center of Cardiovascular Surgery (Moscow, Russia) in 2017 year. The study was performed in accordance with principles of the Declaration of Helsinki. The protocol of the study was approved by local ethics committee of Bakulev Research Center. All patients provided written informed consent to the CT scanning procedures and agreed to data retrieval, analysis and publication.

A patient-specific anatomical model was comprised of a biventricular cardiac model and a homogeneous torso model. Data processing steps included segmentation of the torso and the ventricles CT volume models, generation of the polygonal surface meshes (“Amycard 01 C” software, EP Solutions SA) and creation of tetrahedral final element meshes (“Gmsh” software Geuzaine and Remacle, 2009).

The bidomain model with the strong heart-torso coupling and TNNP cellular model (ten Tusscher et al., 2004) were used for simulation of a myocardium electrical activity. Transmural and apico-basal cellular heterogeneities were simulated using the approaches proposed in Keller et al. (2012) and ten Tusscher and Panfilov (2006), respectively. Simulations of cardiac electrical activity were performed with the methodology described in Ushenin et al. (2017) using the Cardiac CHASTE software (Mirams et al., 2013). In each anatomical patient model, these finite-element calculations resulted in the transmembrane potentials in the myocardial volume, while the electrical potentials were simulated at each node of the tetrahedral meshes. The torso surface potentials were used as the input for testing the proposed inverse routines.

We simulated three focal type electrical activation patterns. The focal origins were in the lateral wall of the left ventricle (LV) for the patient heart 1, in the right ventricular apex (RVA) for the patient heart 2 and in the right ventricular outflow tract (RVOT) for the patient heart 3. A “virtual” rectangular current pulse of −50μ*A* with a duration of 3 ms applied to an area of 6 mm was used for initiation of a cardiac excitation. “Virtual” action potential signals and local unipolar electrograms on the ventricular surface as well as the body surface ECG were obtained as a result of the simulation.

The first part of the evaluation protocol included computation of the ESL (function *w*_1_) from the simulated electrical potential *u*_1_ on the myocardial surface according to the formula (32) and comparison of this function's morphology in space and time with the transmembrane action potentials.

The second part of the evaluation protocol included an actual testing of the proposed algorithms for the solution of the inverse problem. For this, the BSPM were distorted by an additive Gaussian noise of 50 dB SNR and used for reconstruction of the electrical potential on the endocardial and epicardial surface of the ventricles. The reconstructed local unipolar electrograms on the myocardial surface were compared to the references electrograms obtained from the simulations.

We used the following metrics to estimate solution quality:

(40) $$re_{x}=\frac{1}{M}\sum_{i=1}^{M}\frac{\sqrt{\sum_{j=1}^{N_{1}}{\left(u_{1}(x_{j},t_{i})-u_{1}^{num}(x_{j},t_{i})\right)}^{2}}}{\sqrt{\sum_{j=1}^{N_{1}}u_{1}{\left(x_{j},t_{i}\right)}^{2}}}$$

(41) $$\begin{array}{r} cc_{t}=\frac{1}{N_{1}}\overset{N_{1}}{\underset{i=1}{\sum }}cc\left(u_{1}\left(x_{i},t\right),u_{1}^{num}\left(x_{i},t\right)\right), \end{array}$$

where *u*_1_(*x, t*) is the given solution, $u_{1}^{num}\left(x,t\right)$ is the inverse reconstruction, *cc*(·, ·) – is the correlation coefficient, *M* is the number of time instances, *N*_1_ number of nodes in the heart triangular mesh.

The quantity *re*_*x*_ provides the spatial error of the solution for each instant *t*_*i*_, whereas *cc*_*t*_ shows the accuracy of the reconstructed electrogram at each node on the heart mesh. These errors are calculated separately for epicardial and endocardial surfaces of the heart ventricles.

We tested the following inverse numerical scheme:

1. Tikhonov solution of the equation *Au*_1_ = *f*_0_ with the conventional EP transfer matrix *A* and three types of constraints: 0th order $∥u_{1}∥{}_{2}^{2}\equiv ∥I_{11}u_{1}∥{}_{2}^{2}$, 1st order $∥\frac{\partial u_{1}}{\partial n}∥{}_{2}^{2}\equiv ∥Du_{1}∥{}_{L_{2}}$ and 2nd order $∥\Delta_{\Gamma_{1}}u_{1}∥{}_{2}^{2}\equiv ∥Lu_{1}∥{}_{2}^{2}$.
2. Tikhonov solution of the equation 4π*G*_01_*w*_1_ = *f*_0_, *u*_1_ = *G*_11_*w*_1_ with the ESL transfer matrix *G*_01_ and the constraint $∥w_{1}∥{}_{2}^{2}\equiv ∥I_{11}w_{1}∥{}_{2}^{2}$, see Equation (34).
3. Combination of these two approaches, i.e., Tikhonov solution of the equation *Au*_1_ = *f*_0_ with conventional EP matrix *A* but with the new type of constraint $∥w_{1}∥{}_{2}^{2}\equiv ∥G_{11}^{-1}u_{1}∥{}_{2}^{2}$ for the ESL, see Equations (37), (38).

We found the value of the regularization parameter λ thanks to the L-Curve method (Hansen, 2000).

## 3. Results

### 3.1. Properties of the ESL (Function *w*_1_)

**Figure 5:**
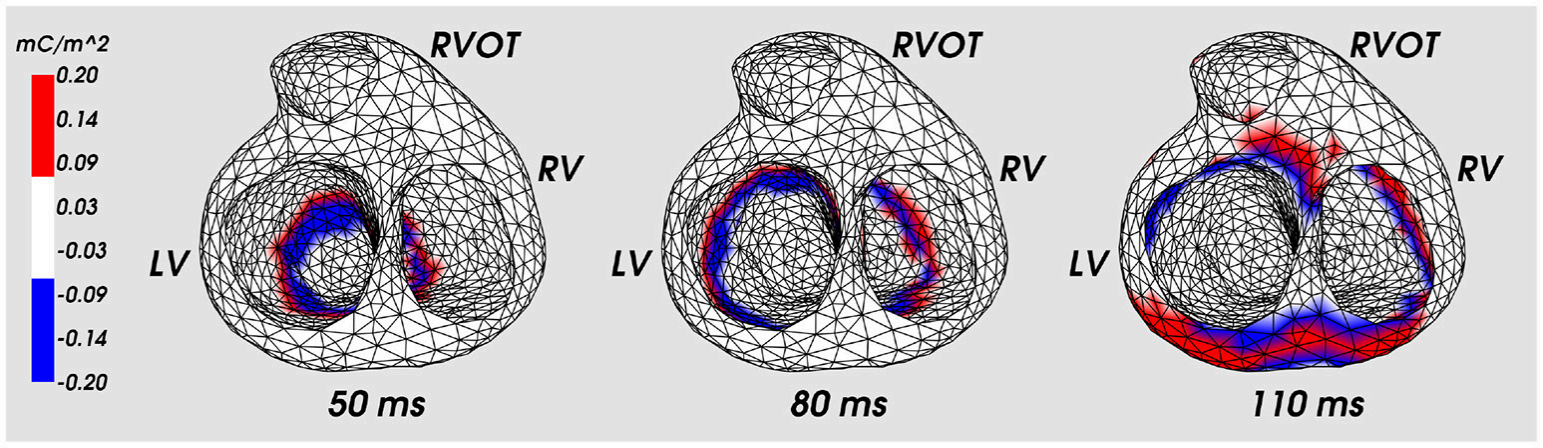
Distribution of the ESL density (function *w*_1_) on the heart surface for the fixed time moments of cardiocycle. Cardiac excitation was initiated in the apical area. LV, left ventricle; RV, right ventricle; RVOT, right ventricle outflow tract.

**Figure 6:**
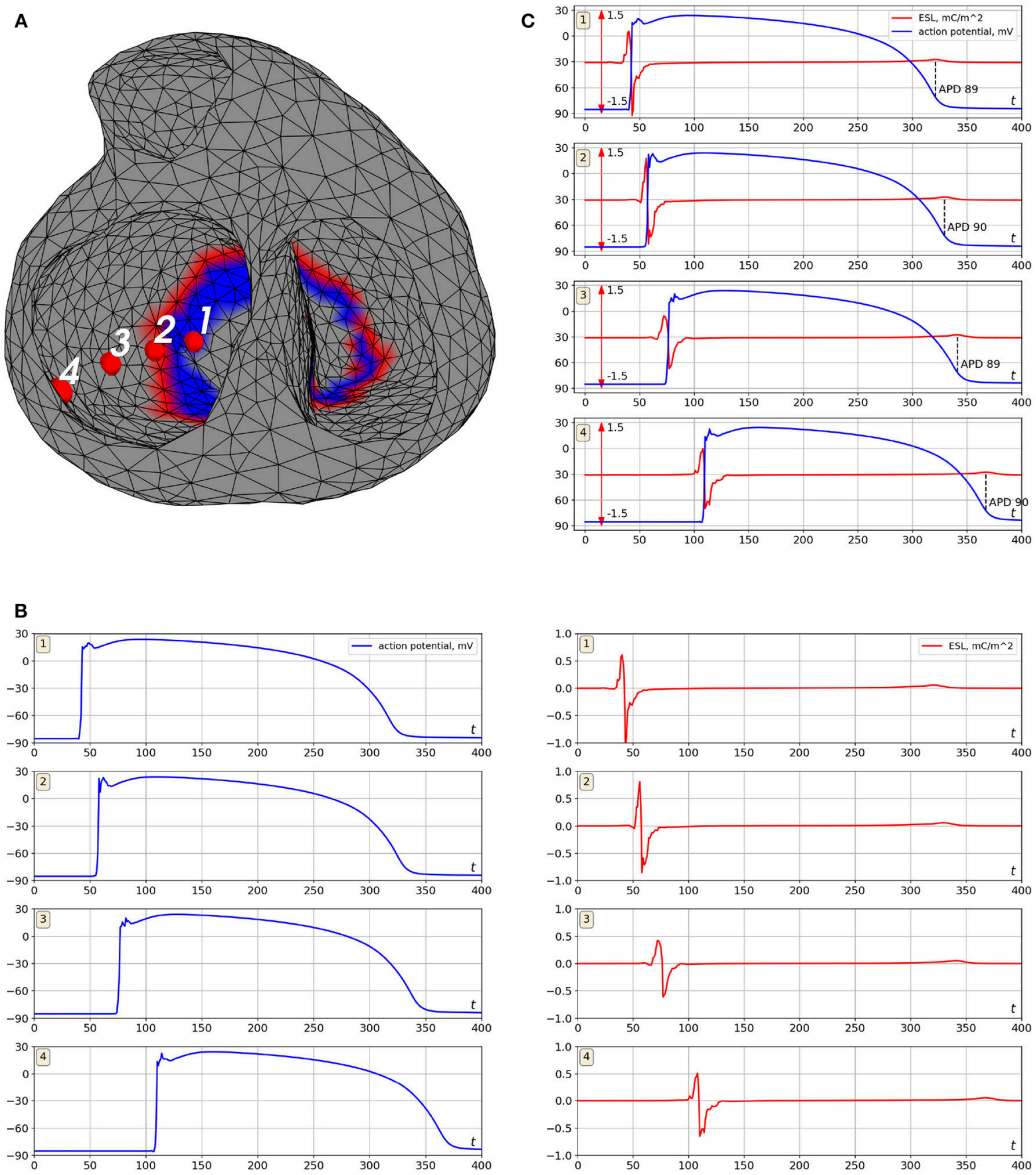
Example of ESL and transmembrane action potential signals (simulation data). Cardiac excitation was initiated in the apical area. **(A)** is locations of the points where the signal was computed, **(B)** is transmembrane action potentials (left panel) and the ESL signals (right panel), **(C)** is merged transmembrane action potentials and ESL signals. Notation *mC*/*m*^2^ is the millicoulomb per square meter, the unit for an electrical charge density.

Figure 5 shows the distribution of the ESL (function *w*_1_(*x, t*)) on the heart surface for several time instants of the cardiocycle. Figure 6 shows ESL as time signals at several points of the ventricular surface. The ESL in space as well as in time domains looks like a sparsed function and well reflects the fronts of myocardial depolarization and repolarization.

The ESL signals shape has a form of bipolar spike associated with time moments of myocardial depolarization and low-amplitude wave associated with myocardium repolarization. Depolarization part of the signal has similar morphology at all points of the myocardial surface with the first peak being positive and the second one negative (see Figure 6B). Duration of depolarization spike varied from 12 to 22 ms, its magnitude varied from 0.5 to 1.2 mC/m^2^.

The repolarization waves were positive in 91% of ESL signals and negative in 2% of the signals. In 7% of the signals the repolarization waves were biphasic with the first negative half-wave. Duration of the repolarization waves varied from 18 to 27 ms, their magnitude varied from 0.05 to 0.2 mC/m^2^.

The zero-crossing value between positive and negative peaks of the signals matched with depolarization time moment (see Figure 6C). The mean difference between that zero point of the single layer density signal and maximal slope of transmembrane action potential (TMP) up-stroke was 1.3±2.4 ms.

The time moment of maximum positive monophasic repolarization waves in ESL signals corresponded to 91±4% level of TMP repolarization (see Figure 6C). Biphasic and negative repolarization waves were also associated with repolarization phase of TMP, but reliable identification of connections between the moment of their appearance and the level of myocardial repolarization requires more data and further analysis. Therefore, ESL signals can be potentially used for detection of depolarization and repolarization of the myocardium. However, development of this method requires further investigations.

### 3.2. Accuracy of the Inverse Solutions

Table 1 shows results of the numerical experiments. The first column in the table shows the type of equation used, the second column shows the regularization constraint and the last columns show the *re*_*x*_ and *cc*_*t*_ metric values (see (40), (41)) separately for the LV, RVA and RVOT simulations and epicardial and endocardial surfaces.

**Table 1:** Results of the inverse reconstruction.

**Equation**	**Reg**	**LV lateral**				**RV apex**				**RVOT**			

		**Epi**		**Endo**		**Epi**		**Endo**		**Epi**		**Endo**	
		*re*_*x*_	*cc*_*t*_	*re*_*x*_	*cc*_*t*_	*re*_*x*_	*cc*_*t*_	*re*_*x*_	*cc*_*t*_	*re*_*x*_	*cc*_*t*_	*re*_*x*_	*cc*_*t*_
**INTERNAL STATEMENT IN TERMS OF EP**													
*Au*_1_ = *f*_0_	$||u_{1}|{|}_{2}^{2}$	6.7e−1	0.89	9.6e−1	0.19	9.0e−1	0.85	9.7e−1	0.12	6.8e−1	0.85	9.5e−1	0.33
*Au*_1_ = *f*_0_	$||\frac{\partial u_{1}}{\partial n}|{|}_{2}^{2}$	6.3e−1	0.91	17.8e−1	0.45	13.8e−1	0.86	27.2e−1	0.24	7.6e−1	0.87	15.9e−1	0.40
*Au*_1_ = *f*_0_	$||Lu_{1}|{|}_{2}^{2}$	6.2e−1	0.91	14.6e−1	0.52	7.8e−1	0.84	19.7e−1	0.32	8.0e−1	0.87	15.7e−1	0.46
**EXTERNAL STATEMENT IN TERMS OF ESL**													
*G*_01_*w*_1_ = *f*_0_	$||w_{1}|{|}_{2}^{2}$	6.2e−1	0.91	4.3e−1	0.83	6.0e−1	0.84	7.6e−1	0.69	7.6e−1	0.87	7.7e−1	0.75
**COMBINED STATEMENT IN TERMS OF EP AND CONSTRAINT ON ESL**													
*Au*_1_ = *f*_0_	$||w_{1}|{|}_{2}^{2}$	6.2e−1	0.91	4.4e−1	0.83	6.1e−1	0.84	7.6e−1	0.69	7.6e−1	0.87	7.8e−1	0.75

**Figure 7:**
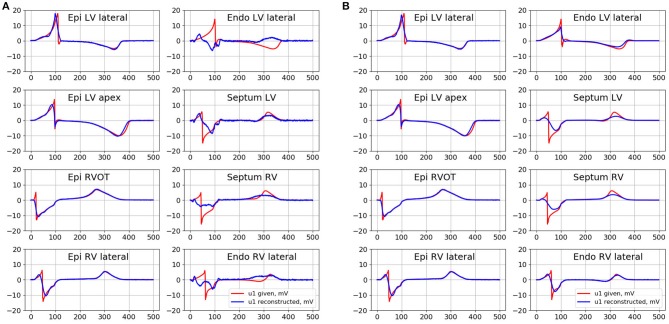
Given (red curves) and inverse reconstructed electrograms (blue curves) in different point of epicardial and endocardial surface for the simulation of the pacing from the RVOT. **(A)** Is the reconstruction with the conventional EP transfer matrix *A* and Tikhonov regularization of 2^nd^ order, **(B)** is the reconstruction with the ESL transfer matrix *G*_01_ and Tikhonov regularization of 0 order.

Electrical signals from the several epicardial and endocardial sites of the ventricles were selected as representative examples in order to provide visual evaluation of their morphology (see Figure 7). The center of LV lateral wall (Epi LV lateral), the apical region (Epi LV apex), anterior-lateral zone of the RVOT (Epi RVOT) and the center of RV lateral wall (Epi RV lateral) were taken on the epicardial surface. The center of LV lateral wall (Endo LV lateral), the central zones of the left (Septum LV) and the right sides (Septum RV) of the ventrical septum and the center of RV lateral wall (Endo RV lateral) were taken on the endocardial surface.

In Figure 7 we present the results of the inverse reconstruction of the electrograms in the defined above points on the heart surface. Figure 7A shows results of reconstruction with the conventional EP transfer matrix *A* and Tikhonov regularization of the 2nd order. Figure 7B shows results of reconstruction with the ESL transfer matrix *G*_01_ and Tikhonov regularization of 0th order.

All algorithms demonstrated similar accuracy on the epicardial surface, but their accuracy on the endocardial surface was significantly different.

On the endocardial surface algorithm in terms of EP with conventional transfer matrix *A* and 0th order regularization demonstrated poor accuracy. The reconstructed electrograms have near-zero magnitude and poor correlation with the references electrograms. Algorithms with the 1st order regularization provided poor accuracy in terms of relative error, but the reconstructed electrograms correlated better with the reference signals (see Table 1). Algorithm with the 2nd order regularization showed slightly better results in comparison to 1^st^ order. However, this algorithm did not allow to reconstruct the electrograms morphology with acceptable quality. In particular, reconstructed electrograms at the Endo LV lateral, RV lateral and Septum RV sites has opposite polarity at QRS part and at the Endo LV lateral site has opposite polarity at ST part than the reference electrograms (see Figure 7A). Detailed metrics values are given in the Table 1.

Both algorithms in terms of ESL significantly improved the accuracy on the endocardial surface. These algorithms showed low relative error and high correlation coefficient. The morphology of endocardial electrograms on the LV and RV lateral walls of the ventricles as well as at RVOT were reconstructed with enough accuracy. The electrograms on the LV and RV ventricular septum were more smoothed, but the basic elements of their morphology (polarities of the electrogram waves) were reconstructed correctly.

Activation and recovery times are commonly used in the clinical practice as one of the important outputs of non-invasive cardiac imaging. Some numerical results in detection of activation and repolarization times from electrograms reconstructed by the proposed ESL algorithm are given in the Supplementary Material 1.

## 4. Discussion

Non-invasive cardiac electrical mapping on both epi- and edocardial surfaces of the heart can provide more detailed information about cardiac electrical activity. However, this methodology is more challenging compared to the non-invasive epicardial mapping. In previous works the problem of endo-epicardial mapping was attacked in two directions. The former was to extend the inverse electrocardiography problem in terms of epicardial potentials to a problem in terms of epicardial and endocardial potentials. The second one was to reconstruct cardiac electrical activity on the epicardium and endocardium in terms of the EDL or in terms of intramural “equivalent” electrical sources related to cardiac transmembrane potentials.

In this article, we introduced a novel representation of cardiac sources in terms of the ESL potential. This approach, in a sense, combines these two directions. Utilizing EDL for representation of cardiac electrical activity was motivated by the following reasons. First, it is well known that the electrical potential on the cardiac surface can be understood as a sum of two components: so-called “near field,” reflecting local myocardial electrical activity and so-called “far field,” which is generated by electrical sources at remote segments of the heart. This fact leads to certain difficulties in interpretation of local unipolar electrograms with respect to depolarization and repolarization times. In contrast, EDL allows detecting the local electrical activity of the myocardium with greater precision. Secondly, EDL signals have higher level of regularity in comparison to local unipolar electrograms. This trait of EDL may provide additional opportunities for regularization of the inverse problem.

Our results of the presented *in-silico* experiments showed that the ESL representation of cardiac electrical activity has also some attractive properties. ESL density correlated well with the local electrical activity of the myocardium. ESL density as time signals can be used for detection of activation and recovery times, calculating activation and recovery intervals and reconstruction of activation and recovery sequences. Note that detection of repolarization sequences by local electrograms meets some methodological difficulties (Cluitmans et al., 2017). However, repolarization abnormalities can be an important substrate of reentrant atrial and ventricular arrhythmias. Therefore, possible application of ESL for detection of repolarization abnormalities seems to be promising.

Moreover, ESL density is a temporally localized function exhibiting very similar morphology for all ventricular sites. These ESL features can be potentially used for narrowing down the set of admissible solutions in construction of regularization methods.

Note, these results were obtained for the myocardial model with isotropic electrical conductivities. The proposed approach for ESL computation does not require assumptions of myocardium anisotropy. Therefore, it can be translated directly to the more realistic anisotropic model of the myocardium. However, ESL been computed this way may slightly differ from the “physical” single layer density in case of the media with anisotropic electrical conductivity. To emphasize this fact, we used the term “equivalent” single layer density (ESL). However, we suppose that investigation of electrophysiological meaning of representation of cardiac electrical activity in form of electrical single layer for the more realistic anisotropic model requires more precise mathematical definition of electrical single layer and more complex algorithm for its computation. We address this task to further research.

The most common discretization method for the inverse potential problem, i.e., for reducing the boundary value problem for Laplace equation to a system of linear algebraic equations is BEM (Yun et al., 1997; Gulrajani, 1998 and description in section 2.1). It this work we proposed an alternative BEM scheme for assembling the transfer matrix, which is closely related to the ESL representation of the cardiac electrical field. Though we considered this method in the context of the endo-epicardial potential inverse problem, this approach can be used for the reconstruction of electrical potentials on the epicardial surface only, in contrast to the EDL.

Moreover, the presented derivation of the ESL transfer matrix allowed to identify the intrinsic structure of the conventional one, that can be split into two matrices: a well-conditioned *G*_11_ and ill-conditioned *G*_01_, whereby their elements are the inverse euclidean distances not depending on the solid angles involved by EDL computations.

A technical benefit of the splitting lies in this simple structure allowing greater numerical precision of the two matrices. In particular, the novel way for construction of the transfer matrix does not require calculation of normal vectors, thus eliminating possible mesh-related artifacts. Furthermore, a simple structure of ill-conditioned matrix *G*_01_ provides an alternative basis for regularization approaches.

The above results were obtained for the simplified torso model with homogeneous electrical conductivities. We suppose that the translation of the reconstruction algorithm to the clinical practice requires more realistic human torso model with different electrical conductivities of the internal organs. In that cases the structure of the transfer matrix for the inverse problem in terms of ESL will be more complex. Identification of its structure is a task for further research.

We also presented a two-step method for solving the inverse potential problem including computation of the ESL density as an intermediate step. This method has some formal similarities with the method of fundamental solutions (MFS). The MFS was proposed for solving the inverse problem in the epicardial statement and showed promising results (Wang and Rudy, 2006). Briefly, MFS is based on computation of values of “virtual” point electrical sources placed outside of the domain of interest and subsequent computation of the cardiac electrical potential as a linear combination of these electrical sources. The MFS also allows usage of meshless construction of the transfer matrix. However, in contrast to the ESL on the myocardial surface, the electrical sources do not have a physiological meaning, i.e., they cannot be used for evaluating the local electrical activity of the myocardium. Next, in contrast to the ESL matrix *G*_11_, the MFS matrix mapping electrical source values to the electrical potential on the cardiac surface is ill-conditioned. Thus, this matrix cannot be used as a regularization operator.

The most interesting and unexpected result was, in our opinion, obtained in the investigation of regularization effects of the ESL density on the heart. We first tested the 0th order Tikhonov regularization for the inverse problem in the ESL statement. Then, we used $G_{11}^{-1}$ as a regularization operator for the conventional EP statement of the inverse problem. We found that both schemes provided significantly more accurate solutions compared to the other Tikhonov regularizations.

Still, the regularizing properties of the inverse single layer operator require further investigation and theoretical explanation. It is worth noting that the ESL regularization increased the accuracy predominately on the endocardial surface of the heart. We can hypothesize that independence of the ESL from solid angles contributed to the accuracy increase for the complex “w-shape” geometry of the endocardial surface. Nevertheless, a detailed mathematical interpretation of the obtained results as well as a more general challenge of developing an optimal regularization operator for the inverse ECG problem is a subject for further investigations.

As our conceptual approach targeted an improved reconstruction of the electrogram itself, further efforts should be undertaken to quantify ESL advantages in estimation of derived parameters, such as activation times, frequency maps etc. The presented work focused solely on a mathematical description of the novel ESL formulation and its simulation-based proof-of-concept, making thorough *in silico* and clinical evaluation needed in order to translate our findings into practical benefits.

## 5. Conclusions

In this article, we proposed a novel statement of the inverse problem of ECG which is based on a representation of the electrical potential on the cardiac surface as ESL on the same surface. The results of *in-silico* experiments using personalized cardiac models demonstrated that the introduced ESL density well correlates with local electrical activity of the myocardium.

The reconstruction method was considered in two basic versions. The first version included assembling of a transfer matrix mapping the ESL density to the body surface potentials using the BEM and solving the matrix equation with Tikhonov regularization of 0th order. The second version used the conventional transfer matrix mapping EP on the cardiac surface to the BSPM and then applied Tikhonov regularization imposing constraints based on the single layer operator on the heart. The results demonstrated that both versions provided more accurate solution on the ventricular endocardial surface compared to the classical approach with Tikhonov regularization of 0th, 1st, and 2nd orders.

The proposed modifications in the solution scheme may improve non-invasive reconstruction of cardiac electrical activities on the endocardial part of the heart.

## 6. Limitations

We used only limited numerical simulations cases for testing feasibility of the proposed method. In our future work, we intend to extensively study performance of the presented approach in both *in silico* and clinical setups. Forward and inverse simulations were performed for a homogeneous torso model with an isotropic heart. While heart anisotropy affects both heart and torso surface potentials, it does not influence the relationship between the ESL source model and EP on the heart surface. The ESL variable obtained this way may slightly differ from the “physical” single layer density in case of the media with anisotropic electrical conductivity. To emphasize this fact, we used the term “equivalent” single layer density.

Only ventricular focal activation patterns were considered in the present work. Further studies should include more complex excitation propagation patterns as well as a comparative analysis between the derived (e.g., activation times) clinically relevant parameters.

## Author Contributions

All authors contributed equally to this manuscript including development of algorithms, data processing and manuscript writing.

### Conflict of Interest Statement

The authors declare that the research was conducted in the absence of any commercial or financial relationships that could be construed as a potential conflict of interest.
