# Supplementary material for: Solving the Inverse Problem of Electrocardiography on the Endocardium Using a Single Layer Source
Source: Front Physiol. 2019 Feb 5;10:58. doi: 10.3389/fphys.2019.00058 (PMC6370732; doi:10.3389/fphys.2019.00058)
Supplement: Supplementary file 1 [file Data_Sheet_1.PDF]

Solving the inverse problem of electrocardiography on the endocardium using a single layer source

Alexander Kalinin [1,*], Danila Potyagaylo [1] and Vitaly Kalinin [1]

[1] *EP Solutions SA, Yverdon-les-Bains, Switzerland*

Correspondence*:
EP Solutions SA, Av. des Sciences 13, Yverdon-les-Bains CH-1400, Switzerland
alec.kalinin@gmail.com

SUPPLEMENTAL MATERIAL 1: ACTIVATION AND RECOVERY TIMES

Activation and recovery times are commonly used in the clinical practice as one of the important output of non-invasive cardiac mapping. In order to estimate the ability to reconstruct activation and recovery times by the proposed equivalent single layer (ESL) algorithm, we provide several evaluation tests.

We used the ESL algorithm in the form

$$4\pi G_{01} w_1 = f_0,$$

where f_0 is the known vector, w_1 is the unknown ESL density and the electrical potential u_1 can be reconstructed as $u_1 = G_{11} w_1$ (see equations (34)–(36) in the main manuscript).

The data set (same as in the paper) includes simulation of three focal activation patterns for patient specific anatomies. The focal origins were in the lateral wall of the left ventricle (LV lateral) for the patient heart 1, in the right ventricular apex (RV apex) for the patient heart 2 and in the right ventricular outflow tract (RVOT) for the patient heart 3. The simulated BSPM were distorted by an additive Gaussian noise of 50 dB SNR and used for reconstruction of the electrical potential.

Activation times were detected as the point of maximum negative derivative of the reference and reconstructed electrograms $-\frac{du_1}{dt}$ of the QRS segment. Recovery times were detected as the point of maximum of the reference and reconstructed electrograms $\frac{du_1}{dt}$ of the T segment of the electrical potential (see e.g. C. Ramanathan et. al. Activation and repolarization of the normal human heart under complete physiological conditions. Proc. Natl. Acad. Sci. 2006; 103(16):6309-14).

To estimate the difference between the reference and reconstructed activation maps we used the following metrics: a) correlation coefficient between reference and reconstructed times (“cc”) and b) mean and standard deviation of time differences (“td”) between the reference and reconstructed times.

Suppl. Table 1 shows results of the numerical experiments. Suppl. Figure 1 shows the reference and reconstructed activation time maps, Suppl. Figure 2 shows the reference and reconstructed recovery time maps.

**Variable**	**LV lateral**		**RV apex**		**RVOT**	
	**cc**	**td, ms**	**cc**	**td, ms**	**cc**	**td, ms**
Activation times	0.98	-2.2 ± 5.8	0.98	-2.8 ± 5.9	0.95	-3.0 ± 8.6
Recovery times	0.97	4.1 ± 7.6	0.89	6.3 ± 14.2	0.94	5.9 ± 12.2

Suppl. Table 1. Reconstruction results for activation and recovery times

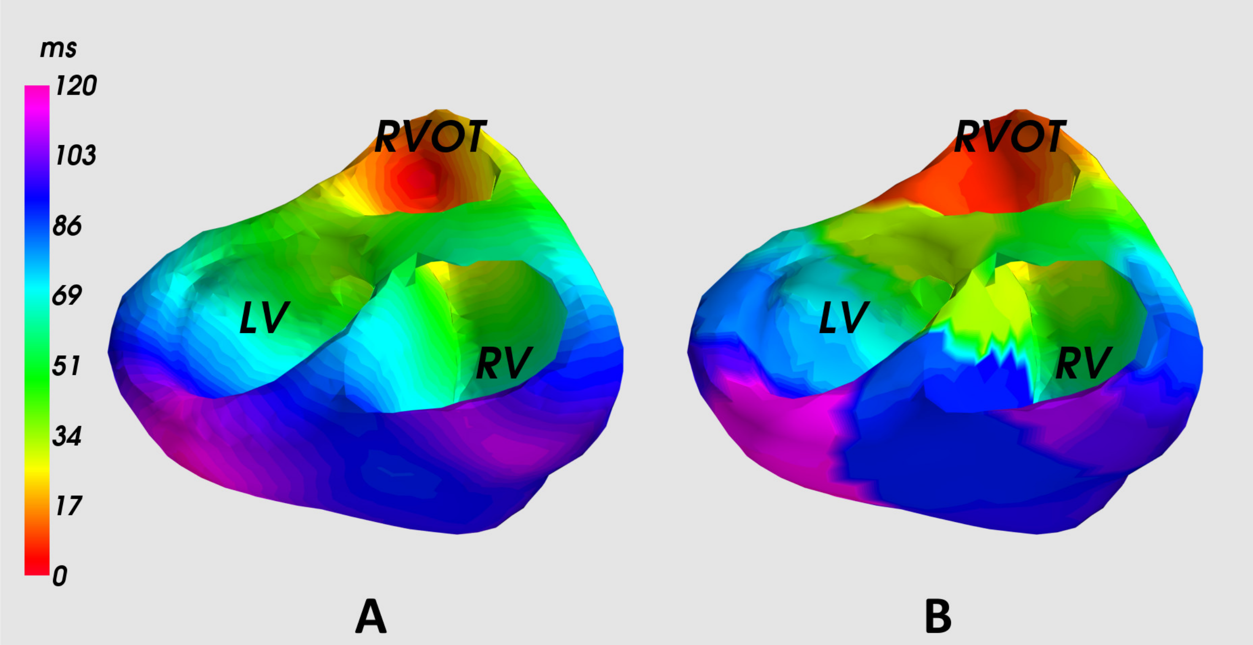

Suppl. Figure 1. Reference (A) and inverse reconstructed (B) activation times for the pacing site in the RVOT.
RVOT, the right ventricle outflow tract, LV, the left ventricle, RV, the right ventricle

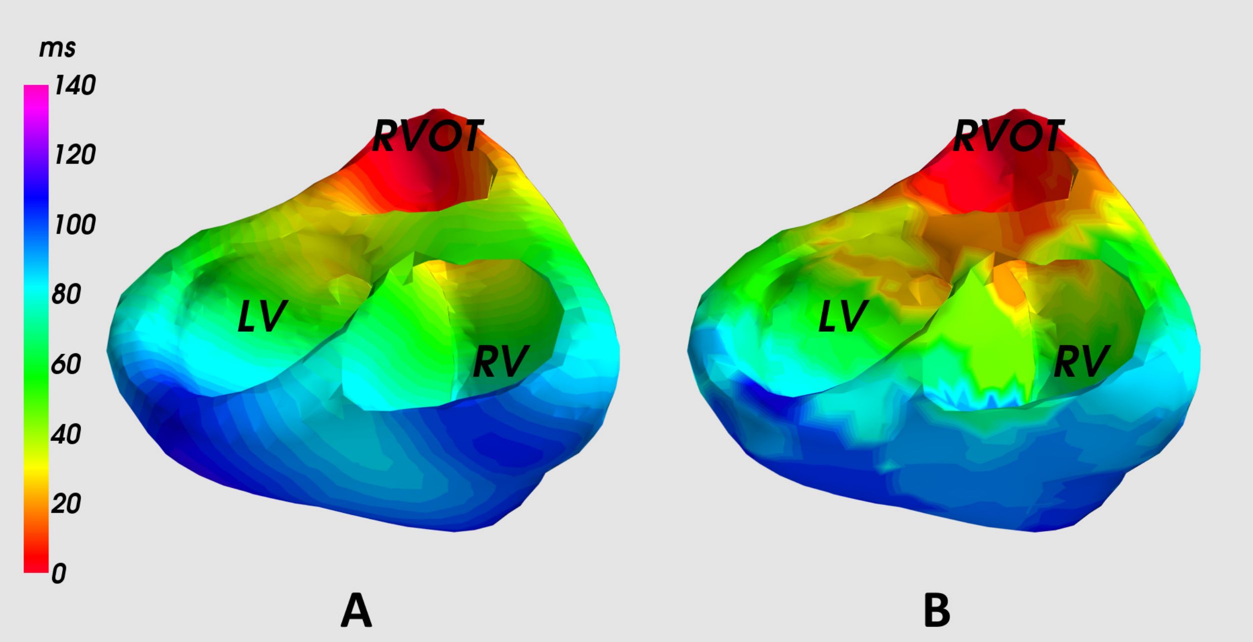

Suppl. Figure 2. Reference (A) and inverse reconstructed (B) recovery times for the pacing site in the RVOT.
RVOT, the right ventricle outflow tract, LV, the left ventricle, RV, the right ventricle
