# Supplementary material for: Solving the Inverse Problem of Electrocardiography on the Endocardium Using a Single Layer Source
Source: Front Physiol. 2019 Feb 5;10:58. doi: 10.3389/fphys.2019.00058 (PMC6370732; doi:10.3389/fphys.2019.00058)
Supplement: Supplementary file 2 [file Data_Sheet_2.pdf]

Solving the inverse problem of electrocardiography on the endocardium using a single layer source

Alexander Kalinin [1,*], **Danila Potyagaylo** [1] **and Vitaly Kalinin** [1]

[1] *EP Solutions SA, Yverdon-les-Bains, Switzerland*

Correspondence*:
EP Solutions SA, Av. des Sciences 13, Yverdon-les-Bains CH-1400, Switzerland
alec.kalinin@gmail.com

APPENDIX A. DISCRETIZATION OF THE BOUNDARY INTEGRAL EQUATION

Let Ω be a domain in the space $\mathbb{R}^3$ bounded by a closed surface Γ_0 from the outside and a closed surface Γ_1 from the inside, see figure 1. For any harmonic function $u(x)$ in the domain Ω we can write the boundary integral equation

$$c(P)u(P) + \int\limits_{\Gamma_0 \cup \Gamma_1} u(Q) \frac{\partial G(P,Q)}{\partial n}\, d\Gamma_Q = \int\limits_{\Gamma_0 \cup \Gamma_1} \frac{\partial u(Q)}{\partial n} G(P,Q)\, d\Gamma_Q, \tag{1}$$

where $P \in \Gamma_0 \cup \Gamma_1$ is an arbitrary fixed point on the domain boundary, $Q \in \Gamma_0 \cup \Gamma_1$ is a point of integration, $c(P)$ is the solid angle, and $G(P,Q) = \frac{1}{|P-Q|}$ is the inverse distance between points P and Q.

Surfaces Γ_0 and Γ_1 are approximated by the triangular meshes $\tilde{\Gamma}_0(N_0, T_0)$ and $\tilde{\Gamma}_1(N_1, T_1)$ where N_0, N_1 are total number of the vertices V_i and T_0, T_1 are number of triangles τ_i (see figure 2).

Every triangle τ_i we call a "boundary element" and the joint mesh $\tilde{\Gamma}_0 \cup \tilde{\Gamma}_1$ is defined as

$$\tilde{\Gamma}_0 \cup \tilde{\Gamma}_1 = \tau_1 \cup \tau_2 \cup \ldots \tau_{T_0} \cup \tau_{T_0+1} \cup \ldots \cup \tau_{T_0+T_1}. \tag{2}$$

Now we introduce the set of linear continuous basis functions $\psi_i(x)$,

$$\psi_i(x) = \begin{cases} 1, & x = V_i, \\ 0, & x = V_j, j \neq i, \\ \text{linear in } \tau_k, V_i \in \tau_k, \end{cases} \tag{3}$$

where $i = 1, 2, \ldots, N_0, N_0 + 1, \ldots, N_0 + N_1$ (see figure 3).

Potential $u(x)$ and its normal derivative are approximated by the series of basis functions $\psi_i(x)$

$$\tilde{u}(x) \approx \sum_{i=1}^{N_0+N_1} a_i \psi_i(x), \quad x \in \tilde{\Gamma}_0 \cup \tilde{\Gamma}_1 \tag{4}$$

$$\frac{\partial \tilde{u}(x)}{\partial n} \approx \sum_{i=1}^{N_0+N_1} b_i \psi_i(x), \quad x \in \tilde{\Gamma}_0 \cup \tilde{\Gamma}_1. \tag{5}$$

Substituting (4) and (5) in (1), we get the discrete version of the boundary integral equation

$$c(V_i)a_i + \sum_{i=1}^{N_0+N_1} a_i \int\limits_{\tilde{\Gamma}_0 \cup \tilde{\Gamma}_1} \psi_i(Q) \frac{\partial G(V_i,Q)}{\partial n}\, d\Gamma_Q = \sum_{i=1}^{N_0+N_1} b_i \int\limits_{\tilde{\Gamma}_0 \cup \tilde{\Gamma}_1} \psi_i(Q) G(V_i,Q)\, d\Gamma_Q \quad (6)$$

and after integration of products of the known basis functions $\psi_i(Q)$ by known inverse distance function $G(V_i, Q)$ and its normal derivative $\frac{\partial G(V_i,Q)}{\partial n}$ we get a system of linear equations

$$\begin{bmatrix} h_{1,1} & \cdots & h_{1,N_0} & h_{1,N_0+1} & \cdots & h_{1,N_0+N_1} \\ \cdots & \cdots & \cdots & \cdots & \cdots & \cdots \\ h_{N_0,1} & \cdots & h_{N_0,N_0} & h_{N_0,N_0+1} & \cdots & h_{N_0,N_0+N_1} \\ h_{N_0+1,1} & \cdots & h_{N_0+1,N_0} & h_{N_0+1,N_0+1} & \cdots & h_{N_0+1,N_0+N_1} \\ \cdots & \cdots & \cdots & \cdots & \cdots & \cdots \\ h_{N_0+N_1,1} & \cdots & h_{N_0+N_1,N_0} & h_{N_0+N_1,N_0+1} & \cdots & h_{N_0+N_1,N_0+N_1} \end{bmatrix} \begin{bmatrix} a_1 \\ \cdots \\ a_{N_0} \\ a_{N_0+1} \\ \cdots \\ a_{N_0+N_1} \end{bmatrix} =$$

$$= \begin{bmatrix} g_{1,1} & \cdots & g_{1,N_0} & g_{1,N_0+1} & \cdots & g_{1,N_0+N_1} \\ \cdots & \cdots & \cdots & \cdots & \cdots & \cdots \\ g_{N_0,1} & \cdots & g_{N_0,N_0} & g_{N_0,N_0+1} & \cdots & g_{N_0,N_0+N_1} \\ g_{N_0+1,1} & \cdots & g_{N_0+1,N_0} & g_{N_0+1,N_0+1} & \cdots & g_{N_0+1,N_0+N_1} \\ \cdots & \cdots & \cdots & \cdots & \cdots & \cdots \\ g_{N_0+N_1,1} & \cdots & g_{N_0+N_1,N_0} & g_{N_0+N_1,N_0+1} & \cdots & g_{N_0+N_1,N_0+N_1} \end{bmatrix} \begin{bmatrix} b_1 \\ \cdots \\ b_{N_0} \\ b_{N_0+1} \\ \cdots \\ b_{N_0+N_1} \end{bmatrix} \quad (7)$$

where

$$g_{i,j} = \sum_l \int\limits_{\tau_l} \psi_j(Q) G(V_i,Q)\, d\tau_Q, \quad l = \{l : V_j \in \tau_l\}, \quad (8)$$

$$h_{i,j} = c(V_i) + \sum_l \int\limits_{\tau_l} \psi_j(Q) \frac{\partial G(V_i,Q)}{\partial n}\, d\tau_Q, \quad l = \{l : V_j \in \tau_l\}. \quad (9)$$

For computation of the integrals in (8) and (9) we use approach described in the papers (Dunavant, 1985; Davey and Hinduja, 1989).

We can write the system of linear equation (7) in a block matrix form

$$\begin{bmatrix} H_{00} & H_{01} \\ H_{10} & H_{11} \end{bmatrix} \begin{bmatrix} u_0 \\ u_1 \end{bmatrix} = \begin{bmatrix} G_{00} & G_{10} \\ G_{10} & G_{11} \end{bmatrix} \begin{bmatrix} 0 \\ q_1 \end{bmatrix} \quad (10)$$

where $u_0 = [a_1, \ldots, a_{N_0}]$ values of the potential $u(x)$ and $q_0 = [b_1, \ldots, b_{N_0}] = 0$ values of the potential normal derivative $\frac{\partial u(x)}{\partial n}$ in the vertices of the mesh $\tilde{\Gamma}_0$; $u_1 = [a_{N_0+1}, \ldots, a_{N_0+N_1}]$ values of the potential $u(x)$ and $q_1 = [b_{N_0+1}, \ldots, b_{N_0+N_1}]$ values of the potential normal derivative $\frac{\partial u(x)}{\partial n}$ in the ventricles of the mesh $\tilde{\Gamma}_1$.

Also we can write system of linear equation (7) as a system of block matrix equation

$$\begin{aligned} H_{00}u_0 + H_{01}u_1 &= G_{01}q_1 \\ H_{10}u_0 + H_{11}u_1 &= G_{11}q_1 \end{aligned} \quad (11)$$

Matrices G_{ij} and H_{ij} have clear physical interpretations. Matrices G_{ij} are the discrete form of the electric single layer integral operator with the sources located on the surface i generating potential on the field surface j. Matrices H_{ij} are the discrete form of the electrical double layer integral operator with the sources located on the surface i generating electrical potential on the field surface j.

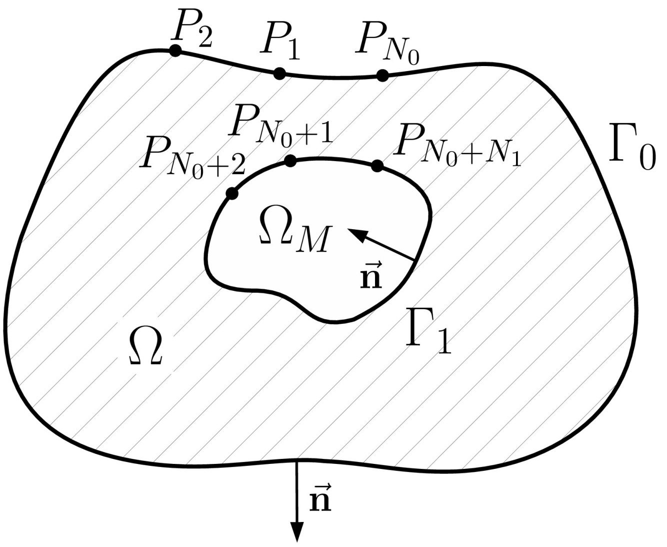

Figure 1. Schematic geometric relationships of the inverse potential problem in the internal statement. Ω is the passive volume conductor domain, Ω_M is the myocardial domain, Γ_0 is the body surface, Γ_1 is the myocardial surface (endo- and epicardial surface), $\vec{\mathbf{n}}$ its unit normal vector directed inward, P_i, $i = 1..N_0 + N_1$ are collocation points used in direct boundary element method, N_0 is the number of collocation points on the Γ_0, N_1 is the number of collocation points on Γ_1.

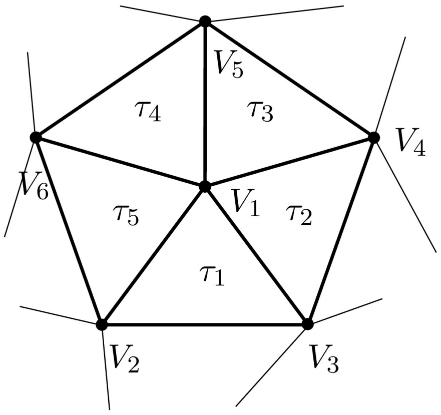

Figure 2. Part of the mesh $\hat{\Gamma}_0$

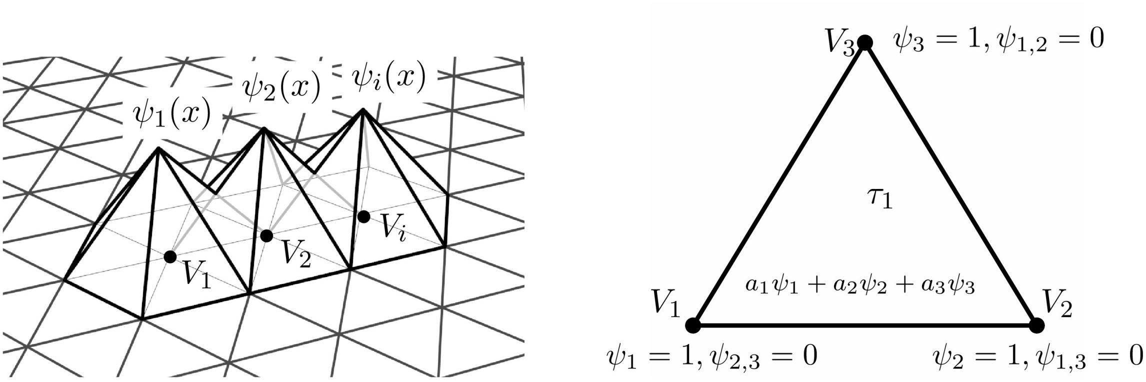

A B

Figure 3. Basis functions on the mesh. **(A)** is continuous linear basis functions ψ, **(B)** is linear approximation in the triangle.
